# Abdominal aortic aneurysm in patients affected by intermittent claudication: prevalence and clinical predictors

**DOI:** 10.1186/1471-2482-12-S1-S17

**Published:** 2012-11-15

**Authors:** Giuseppe Giugliano, Eugenio Laurenzano, Carlo Rengo, Giovanna De Rosa, Linda Brevetti, Anna Sannino, Cinzia Perrino, Lorenzo Chiariotti, Gabriele Giacomo Schiattarella, Federica Serino, Marco Ferrone, Fernando Scudiero, Andreina Carbone, Antonio Sorropago, Bruno Amato, Bruno Trimarco, Giovanni Esposito

**Affiliations:** 1 Department of Clinical Medicine and Cardiovascular and Immunological Sciences, “Federico II” University”, via Pansini 5, 80131, Naples, Italy; 2 Department of Biology and Cellular and Molecular Pathology, Federico II University, via Pansini 5, 80131, Naples, Italy; 3 Department of General, Geriatric, Oncologic Surgery and Advanced Technologies, “Federico II” University”, via Pansini 5, 80131, Naples, Italy

## Abstract

**Background:**

Abdominal aortic aneurysm (AAA) is a frequent cause of death among elderly. Patients affected by lower extremity peripheral arterial disease (LE-PAD) seem to be particularly at high risk for AAA. We aimed this study at assessing the prevalence and the clinical predictors of the presence of AAA in a homogeneous cohort of LE-PAD patients affected by intermittent claudication.

**Methods:**

We performed an abdominal ultrasound in 213 consecutive patients with documented LE-PAD (ankle/brachial index ≤0.90) attending our outpatient clinic for intermittent claudication. For each patient we registered cardiovascular risk factors and comorbidities, and measured neutrophil count.

**Results:**

The ultrasound was inconclusive in 3 patients (1.4%), thus 210 patients (169 males, 41 females, mean age 65.9 ± 9.8 yr) entered the study. Overall, AAA was present in 19 patients (9.0%), with a not significant higher prevalence in men than in women (10.1% vs 4.9%, p = 0.300). Patients with AAA were older (71.2 ± 7.0 vs 65.4 ± 9.9 years, p = 0.015), were more likely to have hypertension (94.7% vs 71.2%, p = 0.027), and greater neutrophil count (5.5 [4.5 – 6.2] vs 4.1 [3.2 – 5.5] x10^3^/μL, p = 0.010). Importantly, the c-statistic for neutrophil count (0.73, 95% CI 0.60 – 0.86, p =0.010) was higher than that for age (0.67, CI 0.56–0.78, p = 0.017). The prevalence of AAA in claudicant patients with a neutrophil count ≥ 5.1 x10^3^/μL (cut-off identified at ROC analysis) was as high as 29.0%.

**Conclusions:**

Prevalence of AAA in claudicant patients is much higher than that reported in the general population. Ultrasound screening should be considered in these patients, especially in those with an elevated neutrophil count.

## Introduction

Systemic atherosclerosis represents the leading cause of morbidity and mortality in the western countries [1-3], with increased prevalence in the elderly population [4,5]. The atherosclerotic disease may involved different part of vascular tree, in particular coronary arteries, carotid arteries and peripheral arteries [6,7] .

Abdominal aortic aneurysm (AAA) is a frequent cause of death in the elderly and its incidence has increased during last decades because of the increasing life-expectancy and the development of easy and low-cost diagnostic tools like ultrasound [5,8-12]*.* The rising incidence of AAA and the severe prognosis in case of rupture with a mortality rate that can be as high as 90% [13] call for early identification and elective repair. However, opposing views have been published on the importance of ultrasound screening for AAA and there is still debate on the high-risk populations who need to be screened [14,15].

Lower extremity peripheral arterial disease (LE-PAD), one of the main expressions of atherosclerosis, affects about 27 million people in Europe and the United States [16] and is associated with a high risk of developing fatal and non-fatal ischemic cardiovascular events [2,17-19]. Patients affected by LE-PAD seem to be at particularly high risk for AAA development [20-22]. Accordingly, we aimed this study at assessing the prevalence and the clinical predictors of the presence of AAA in a homogeneous cohort of LE-PAD patients affected by intermittent claudication, the most frequent clinical expression of LE-PAD.

## Methods

We performed an abdominal ultrasound in 213 consecutive patients with documented LE-PAD attending our outpatient complaining intermittent claudication. Using an Image Point Hx ultrasound system (Hewlett Packard) and a 3.0 MHz transducer, we measured in each patient both antero-posterior and transverse outer diameters at the largest portion of the infrarenal abdominal aorta. AAA was defined by an infrarenal abdominal aorta diameter ≥ 3 cm [23]. Infrarenal abdominal aortas 2.6 to 2.9 cm were defined ectatic.

The diagnosis of PAD was based on the presence of an ankle brachial index (ABI) ≤ 0.90. ABI was measured after participants had rested supine for 5 minutes. The systolic blood pressure in both brachial arteries, and the ankle systolic blood pressure in the right and left posterior tibial and dorsalis pedis arteries were measured using a Doppler probe. The ABI for each leg was then determined using the higher of the two readings from either the posterior tibial or dorsalis pedis arteries, and the higher of the two brachial readings. The lower ABI of the two legs was used for diagnostic purposes.

In each patient, clinical history and risk factors were assessed. Smokers included current and former smokers. Hypertension was diagnosed if systolic arterial pressure exceeded 140 mmHg and/or diastolic arterial pressure exceeded 90 mmHg, or if the patient used antihypertensive drugs. Hypercholesterolemia was diagnosed if plasma total cholesterol exceeded 200 mg/dL, plasma low-density lipoprotein cholesterol exceeded 130 mg/dL, or if the patient used lipid-lowering drugs because of a history of hypercholesterolemia. Diabetes mellitus was diagnosed if plasma fasting glucose exceeded 126 mg/dL or if the patient used hypoglycaemic agents. A history of coronary artery disease, previous myocardial infarction, or ischemic stroke was documented by hospital records. All patients underwent ultrasound examination of carotid arteries.

All participants gave written informed consent to the study, which was approved by our institutional Ethics Committee.

### Blood samples and laboratory assay

Blood was drawn from an antecubital vein using a 19-gauge needle in a Vacutainer system (Becton-Dickson, Franklin Lakes, NJ). Neutrophil count was measured with the Bayer H*2 hematology analyzer (Bayer Diagnostic Division, Tarrytown, NY).

### Statistical analysis

Statistical analyses were performed using SPSS 12.0 (SPSS, Inc., Chicago, IL, USA). Variables were expressed as absolute numbers and percentage or mean ± SD, with the exception of neutrophil count, which was expressed as median and inter-quartile range because of their skewed distribution. Comparisons were made by t-test for unpaired samples, χ^2^ test, or Mann–Whitney U test, as appropriate. For continuous variables, receiver-operating characteristic (ROC) curve analysis was used to identify the threshold levels that provided the best cut-off for the prediction of the presence AAA. Correlations between continuous variables were evaluated by Spearman analysis.

All statistical tests were two-sided. For all tests, a p-value <0.05 was considered statistically significant.

## Results

**Figure 1:**
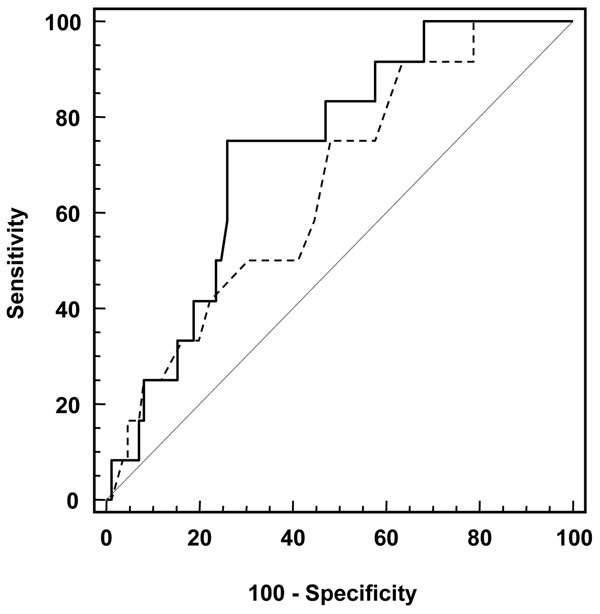


The ultrasound was inconclusive in 3 patients (1.4%), thus 210 patients (169 males, 41 females, mean age 65.9 ± 9.8 yr) completed the study. Table 1 shows baseline characteristics of the study population. Overall, AAA was present in 19 patients (9.0%), with a not significant higher prevalence in men than in women (10.1% vs 4.9%, p = 0.300). Furthermore, patients with AAA were older (71.2 ± 7.0 vs 65.4 ± 9.9 years, p = 0.015), were more likely to have hypertension (94.7% vs 71.2%, p = 0.027), and greater neutrophil count (5.5 [4.5 – 6.2] vs 4.1 [3.2 – 5.5] x10^3^/μL, p = 0.010) (Table 2). The prevalence progressively increased with age (p for trend = 0.013), with a maximum of 15.8% in over 75-year-subjects (Table 3). Of note, in our population we observed a higher prevalence of carotid stenosis >50% in claudicant patients with AAA, although not significant (47.4 vs 29.3%, p = 0.105). No relationship was found between the ankle/brachial index and the presence of AAA. For continuous variables, which are associated to the presence of AAA at univariate analysis (i.e. age and neutrophil count), ROC curve analysis was performed to evaluate the best cut-offs to predict the presence of the disease (Figure 1). The best cut-offs were ≥ 66 years for age and ≥ 5.1 x10^3^/μL for neutrophil count. Importantly, the c-statistic for neutrophil count (0.73, 95% CI 0.60 – 0.86, p = 0.010) was higher than that for age (0.67, CI 0.56–0.78, p = 0.017) (Figure 1). In our population the prevalence of AAA in claudicant patients with a neutrophil count ≥ 5.1 x10^3^/μL (cut-off identified at ROC analysis) was as high as 29.0%. Of note, Spearman analysis showed that neutrophil count and age are not correlated (ρ = -0.123, p = 0.230).

**Table 1:** Baseline characteristics of the study population (n=210)

	Number	Percentage or mean (SD) or median [25^th^-75^th^ percentiles]
Age (years)		65.9 (9.8)
Males	169	80.5
Height (cm)		167.4 (7.5)
Weight (Kg)		73.9 (11.7)
***Risk factors***		
Smoking	188	89.5
Hypertension	154	73.3
Hypercholesterolemia	137	65.2
Diabetes mellitus	75	35.7
BMI (kg/m^2^)		26.2 (3.4)
WC (cm)		97.6 (9.4)
Metabolic syndrome	96	45.7
***Comorbidity***		
CAD	106	50.5
Previous MI	76	36.2
Previous stroke	8	3.8
***Medications***		
Antiplatelets	191	91.0
Beta blockers	47	22.4
ACE-inhibitors	104	49.5
Statins	130	61.9
***Carotid status***		
Carotid stenosis >50%	65	31
***LE-PAD severity***		
Bilateral LE-PAD	131	62.4
ABI		0.69 (0.22)
***Aortic features***		
Aortic diameter (mm)		20.7 (6.8)
Ectatic aorta	13	6.2
AAA	19	9.0
***Inflammatory status***		
Neutrophil count (x10^3^/μL)		4.4 [3.3-5.7]

SD = standard deviation; BMI = body mass index; WC = waist circumference; CAD = coronary artery disease; MI = myocardial infarction; ACE = angiotensin converting enzyme; LE-PAD = lower extremity-peripheral arterial disease; ABI = ankle/brachial index; AAA = abdominal aortic aneurysm.

**Table 2:** Characteristics of claudicants according to the presence or absence of AAA.

	AAA	No AAA	p
	(n = 19)	(n = 191)	
Age (years)	71.2 ± 7.0	65.4 ± 9.9	0.015
Age ≥ 65 years	16 (84.2)	110 (57.6)	0.024
Males	17 (89.5)	152 (79.6)	0.300
***Risk factors***			
Smoking	18 (94.7)	170 (89.0)	0.437
Hypertension	18 (94.7)	136 (71.2)	0.027
Hypercholesterolemia	15 (78.9)	122 (63.9)	0.188
Diabetes Mellitus	4 (21.1)	71 (37.2)	0.162
BMI (kg/m^2^)	26.5 ± 3.7	26.2 ± 3.4	0.742
WC (cm)	96.7 ± 9.6	97.7 ± 9.4	0.708
Metabolic syndrome	9 (47.4)	87 (45.5)	0.879
***Comorbidity***			
CAD	10 (52.6)	96 (50.3)	0.844
Previous MI	5 (26.3)	71 (37.2)	0.348
Previous stroke	1 (5.2)	7 (3.7)	0.729
***Medications***			
Antiplatelets	18 (94.7)	173 (90.6)	0.547
Beta blockers	2 (10.5)	45 (23.6)	0.194
ACE-inhibitors	11 (57.9)	93 (48.7)	0.444
Statins	10 (52.6)	120 (62.8)	0.383
**Carotid status**			
Carotid stenosis >50%	9 (47.4)	56 (29.3)	0.105
***LE-PAD severity***			
Bilateral LE-PAD	13 (68.4)	118 (61.8)	0.569
ABI	0.64 ± 0.11	0.69 ± 0.23	0.315
***Inflammatory status***			
Neutrophil count (x10^3^/μL)	5.5 [4.5 – 6.2]	4.1 [3.2 – 5.5]	0.010

Values are n (%) or mean ± SD or median [interquartile range].

AAA = abdominal aortic aneurysm; BMI = body mass index; WC = waist circumference; CAD = coronary artery disease; MI = myocardial infarction; ACE = angiotensin converting enzyme; LE-PAD = lower extremity-peripheral arterial disease; ABI = ankle/brachial index; SD = standard deviation.

**Table 3:** Distribution of AAA by age.

	Total	AAA	Prevalence
Age < 55 years	25	0	0%
Age 55 – 64 years	59	3	5.1%
Age 65 – 74 years	88	10	11.4%
Age ≥ 75 years	38	6	15.8%

p for trend = 0.013

## Discussion

The present study demonstrates that prevalence of AAA in claudicant patients is much higher than that reported in the general population, with the highest clinical predictors being advanced age and an elevated inflammatory status.

To decrease the number of deaths from ruptured AAA, early detection by screening subjects at high risk is needed. Physical examination is not effective in identifying the presence of an AAA, while abdominal ultrasound is an accurate test to detect the presence of this life-threatening disease [23]. Elderly males are those who show the highest prevalence of the disease in the general population. Indeed there is evidence that screening programs involving males with > 65 years are cost-effective [14,24,25]. The strong association with age has been confirmed also in our population of claudicant patients. Indeed, we observed that the prevalence of AAA dramatically increases after 65 years (optimal cut-off identified at ROC analysis being ≥ 66 years) and no AAA was found in patients younger than 55 years of age. Thus age cut-off to initiate screening for AAA in patients with intermittent claudication seems to be the same than in general population. These results might suggest that performing an abdominal ultrasound in all claudicant patients may not be advisable. However, in our study, we found that an elevated inflammatory status, evaluated by neutrophil count, is a powerful predictor of the presence of AAA in patients with intermittent claudication, and that the neutrophil count did not correlate with age. Initiating the screening in patients ≥ 65 years with intermittent claudication might consequently lead to miss some diagnosis of AAA, and therefore in claudicant patients with an elevated inflammatory status the ultrasound screening should be performed irrespective of age. At this regard, pathophysiological studies are needed to evaluate whether the increased inflammation observed in our population of intermittent claudication patients affected by AAA is a cause or a consequence of the presence of AAA [19,26,27].

## Conclusions

The high prevalence of AAA among patients affected by intermittent claudication makes them a suitable target population for AAA screening. In our opinion ultrasound screening should be considered in these patients, especially in those with an elevated neutrophil count. However, further studies are warranted to elucidate if screening all patients with intermittent claudication for of this life-threatening condition is cost-effective.

## List of abbreviations

LE-PAD: Lower Extremity Peripheral Arterial Disease; AAA: Abdominal Aortic Aneurysm; ABI: ankle/brachial index.

## Competing interests

The authors declare that they have no competing interests.

## Authors’ contributions

GG, EL, CR, GDR, LB, AS, CP, LC, GGS, FS, AC, AS: conception and design, interpretation of data, given final approval of the version to be published, BA, BT: critical revision, interpretation of data, given final approval of the version to be published, GE: conception and design, critical revision, given final approval of the version to be published.

## References

[B1] Morrow DA Braunwald E Bonaca MP Ameriso SF Dalby AJ Fish MP Fox KA Lipka LJ Liu X Nicolau JC Vorapaxar in the secondary prevention of atherothrombotic events The New England journal of medicine 2012 366 1404 1413 10.1056/NEJMoa1200933 22443427

[B2] Giugliano G Di Serafino L Perrino C Schiano V Laurenzano E Cassese S De Laurentis M Schiattarella GG Brevetti L Sannino A Effects of successful percutaneous lower extremity revascularization on cardiovascular outcome in patients with peripheral arterial disease International journal of cardiology 2012 10.1016/j.ijcard.2012.06.055 22790191

[B3] Cassese S Esposito G Mauro C Varbella F Carraturo A Montinaro A Cirillo P Galasso G Rapacciuolo A Piscione F MGUard versus bAre-metal stents plus manual thRombectomy in ST-elevation myocarDial infarction pAtieNts-(GUARDIAN) trial: study design and rationale Catheterization and cardiovascular interventions : official journal of the Society for Cardiac Angiography & Interventions 2012 79 1118 1126 10.1002/ccd.23405 22105879

[B4] Contaldi C Losi MA Rapacciuolo A Prastaro M Lombardi R Parisi V Parrella LS Di Nardo C Giamundo A Puglia R Percutaneous treatment of patients with heart diseases: selection, guidance and follow-up. A review Cardiovascular ultrasound 2012 10 16 10.1186/1476-7120-10-16 22452829 PMC3364155

[B5] Schiano V Sirico G Giugliano G Laurenzano E Brevetti L Perrino C Brevetti G Esposito G Femoral plaque echogenicity and cardiovascular risk in claudicants JACC Cardiovascular imaging 2012 5 348 357 10.1016/j.jcmg.2012.01.011 22498323

[B6] Mannacio V Di Tommaso L De Amicis V Musumeci F Stassano P Serial evaluation of flow in single or arterial Y-grafts to the left coronary artery The Annals of thoracic surgery 2011 92 1712 1718 10.1016/j.athoracsur.2011.05.092 21937019

[B7] Amato B Markabaoui AK Piscitelli V Mastrobuoni G Persico F Iuliano G Masone S Persico G Carotid endarterectomy under local anesthesia in elderly: is it worthwhile? Acta bio-medica : Atenei Parmensis 2005 76 Suppl 1 64 68 16450515

[B8] Cornuz J Sidoti C Pinto Tevaearai H Egger M Risk factors for asymptomatic abdominal aortic aneurysm: systematic review and meta-analysis of population-based screening studies Eur J Public Health 2004 14 343 349 10.1093/eurpub/14.4.343 15542867

[B9] Earnshaw JJ Shaw E Whyman MR Poskitt KR Heather BP Screening for abdominal aortic aneurysms in men BMJ 2004 328 1122 1124 10.1136/bmj.328.7448.1122 15130983 PMC406329

[B10] Hirsch AT Haskal ZJ Hertzer NR Bakal CW Creager MA Halperin JL Hiratzka LF Murphy WR Olin JW Puschett JB ACC/AHA 2005 Practice Guidelines for the management of patients with peripheral arterial disease (lower extremity, renal, mesenteric, and abdominal aortic): a collaborative report from the American Association for Vascular Surgery/Society for Vascular Surgery, Society for Cardiovascular Angiography and Interventions, Society for Vascular Medicine and Biology, Society of Interventional Radiology, and the ACC/AHA Task Force on Practice Guidelines (Writing Committee to Develop Guidelines for the Management of Patients With Peripheral Arterial Disease): endorsed by the American Association of Cardiovascular and Pulmonary Rehabilitation; National Heart, Lung, and Blood Institute; Society for Vascular Nursing; TransAtlantic Inter-Society Consensus; and Vascular Disease Foundation Circulation 2006 113 e463 654 16549646 10.1161/CIRCULATIONAHA.106.174526

[B11] Perrino C Scudiero L Petretta MP Schiattarella GG De Laurentis M Ilardi F Magliulo F Carotenuto G Esposito G Total occlusion of the abdominal aorta in a patient with renal failure and refractory hypertension: a case report Monaldi Arch Chest Dis 2011 76 43 46 21751737 10.4081/monaldi.2011.205

[B12] Cacciatore F Abete P Maggi S Luchetti G Calabrese C Viati L Leosco D Ferrara N Vitale DF Rengo F Disability and 6-year mortality in elderly population. Role of visual impairment Aging clinical and experimental research 2004 16 382 388 15636464 10.1007/BF03324568

[B13] Prance SE Wilson YG Cosgrove CM Walker AJ Wilkins DC Ashley S Ruptured abdominal aortic aneurysms: selecting patients for surgery Eur J Vasc Endovasc Surg 1999 17 129 132 10.1053/ejvs.1998.0718 10063407

[B14] Bergqvist D Bjorck M Wanhainen A Abdominal aortic aneurysm--to screen or not to screen Eur J Vasc Endovasc Surg 2008 35 13 18 10.1016/j.ejvs.2007.06.012 17905605

[B15] Esposito G Franzone A Cassese S Schiattarella GG Capretti G Pironti G Di Serafino L Perrino C Piscione F Chiariello M Endovascular repair for isolated iliac artery aneurysms: case report and review of the current literature J Cardiovasc Med (Hagerstown) 2009 10 861 865 10.2459/JCM.0b013e32832e1904 19543108

[B16] Weitz JI Byrne J Clagett GP Farkouh ME Porter JM Sackett DL Strandness DE Jr. Taylor LM Diagnosis and treatment of chronic arterial insufficiency of the lower extremities: a critical review Circulation 1996 94 3026 3049 10.1161/01.CIR.94.11.3026 8941154

[B17] Meijer WT Hoes AW Rutgers D Bots ML Hofman A Grobbee DE Peripheral arterial disease in the elderly: The Rotterdam Study Arterioscler Thromb Vasc Biol 1998 18 185 192 10.1161/01.ATV.18.2.185 9484982

[B18] Hirsch AT Criqui MH Treat-Jacobson D Regensteiner JG Creager MA Olin JW Krook SH Hunninghake DB Comerota AJ Walsh ME Peripheral arterial disease detection, awareness, and treatment in primary care JAMA 2001 286 1317 1324 10.1001/jama.286.11.1317 11560536

[B19] Brevetti G Giugliano G Brevetti L Hiatt WR Inflammation in peripheral artery disease Circulation 122 1862 1875 21041698 10.1161/CIRCULATIONAHA.109.918417

[B20] Galland RB Simmons MJ Torrie EP Prevalence of abdominal aortic aneurysm in patients with occlusive peripheral vascular disease Br J Surg 1991 78 1259 1260 10.1002/bjs.1800781036 1959001

[B21] Romano M Mainenti PP Imbriaco M Amato B Markabaoui K Tamburrini O Salvatore M Multidetector row CT angiography of the abdominal aorta and lower extremities in patients with peripheral arterial occlusive disease: diagnostic accuracy and interobserver agreement Eur J Radiol 2004 50 303 308 10.1016/S0720-048X(03)00118-9 15145492

[B22] Romano M Amato B Markabaoui K Tamburrini O Salvatore M Follow-up of patients with previous vascular interventions: role of multidetector row computed tomographic angiography of the abdominal aorta and lower extremities The Journal of cardiovascular surgery 2004 45 89 91 15041947

[B23] Fleming C Whitlock EP Beil TL Lederle FA Screening for abdominal aortic aneurysm: a best-evidence systematic review for the U.S. Preventive Services Task Force Ann Intern Med 2005 142 203 211 15684209 10.7326/0003-4819-142-3-200502010-00012

[B24] Amato B Iuliano GP Markabauoi AK Piscitelli V Masone S Compagna R Esposito G Piscione F Endovascular procedures in critical leg ischemia of elderly patients Acta bio-medica : Atenei Parmensis 2005 76 Suppl 1 11 15 16450500

[B25] Galasso G Piscione F Furbatto F Leosco D Pierri A Rosa RD Cirillo P Rapacciuolo A Esposito G Chiariello M Abciximab in elderly with acute coronary syndrome invasively treated: effect on outcome International journal of cardiology 2008 130 380 385 10.1016/j.ijcard.2008.02.015 18590933

[B26] Esposito G Perrino C Schiattarella GG Belardo L di Pietro E Franzone A Capretti G Gargiulo G Pironti G Cannavo A Induction of mitogen-activated protein kinases is proportional to the amount of pressure overload Hypertension 2010 55 137 143 10.1161/HYPERTENSIONAHA.109.135467 19901160

[B27] Giugliano G Brevetti G Lanero S Schiano V Laurenzano E Chiariello M Leukocyte count in peripheral arterial disease: A simple, reliable, inexpensive approach to cardiovascular risk prediction Atherosclerosis 2010 210 288 293 10.1016/j.atherosclerosis.2009.11.009 19963213

